# Hyperspectral imaging and pulse characterization

**DOI:** 10.1038/s41377-022-00964-9

**Published:** 2022-09-13

**Authors:** Spencer W. Jolly

**Affiliations:** Service Opera-Photonique, Université libre de Bruxelles (ULB), Brussels, Belgium

**Keywords:** Imaging and sensing, Ultrafast photonics

## Abstract

An advanced method for hyperspectral imaging was combined with phase retrieval and standard pulse characterization techniques to characterize ultrashort laser pulses and ultrashort processes to a new level of precision in a single shot.

Dispersive optics and optical systems can impart spatially-varying chromatic phase or amplitude terms on an ultrashort pulse such that the space and time degrees of freedom can no longer be treated separately. Such correlations, called space-time couplings (STCs) have been known for decades to be relevant in commonplace optics such as lenses or prisms^1^. However, more recently the need to measure arbitrary STCs in ultrashort pulses is becoming more urgent, with significant development taking place on improving characterization devices^2,3^. Importantly, spatio-temporal characterization techniques require measuring both the space-time amplitude and phase of an ultrashort electromagnetic pulse, which is a complex 3D data cube.

The need for high performing devices is based on three inter-related use cases: (1) to assess the performance and alignment of cutting-edge laser systems (especially CPA systems), (2) to confirm the success or assess the performance of spatio-temporal shaping, and (3) to measure the space-time effects of a light-matter interaction experiment. Every application will have its own requirements on the device, but in general the higher the resolution in space and time/frequency the better. And more importantly, measuring the entire space-time field of an ultrashort pulse may be necessary in a single-shot either due to the laser having a low repetition-rate, or the process being extremely transient.

Single-shot devices have been demonstrated, for example the STRIPED-FISH technique^4^. In this technique different colors are multiplexed on a 2D detector and spatially-interfere separately with a known STC-free reference. This allows for measurement of both the spatial amplitude and phase of the multiplexed frequencies, which can be reconstructed in time assuming that the reference is perfect. However, the frequency resolution depends on how many colors you can multiplex at once on the detector, and the more highly you resolve the frequencies the less you will resolve them in space due to the finite size of the detector. These limitations on resolution are a commonly accepted price to pay for a single-shot device. STRIPED-FISH was recently applied to a low rep-rate Terawatt laser system^5^, showing it’s ability to measure a complex unknown pulse, and even show the fluctuations of STCs over time—possible only due to its single shot capability.

Now, writing in this issue of *Light: Science & Applications*, a team of scientists from the Huazhong University of Science and Technology in China along with colleagues from other institutes in China, Canada, and the U.S. has successfully implemented a technique that measures in a single-shot without similar constraints on the resolution^6^. Their technique, referred to as compressed optical field topography (COFT) borrows advanced techniques and algorithms from the field of hyperspectral imaging, and combines it with phase-retrieval and spectral phase characterization that are well known to ultrafast optics practitioners. Beyond the general technique, they show two different ways to implement it, and use COFT to measure the space-time dynamics in an ultrafast air-plasma experiment.

One way to implement COFT is shown in Fig. 1. In this implementation they essentially do three measurements in one shot using two devices, a CCD camera and a Frequency-resolved Optical Gating device (FROG)^7^. On the CCD camera they measure both the near-field (unfocused) and far-field (focused) intensity profiles, and with the FROG they can resolve the spectral phase at one sample position in the near-field beam. However, the key components before the CCD camera are a so-called coded aperture and a dispersive element (a prism in this case). This system seemingly scrambles the CCD images, but it actually composes an advanced technique called Coded-Aperture Snapshot Spectral Imaging (CASSI)^8^—this is where hyperspectral imaging comes in. With the unscrambled intensity profiles one can perform phase-retrieval to calculate the spatial phase (wavefront) of the beam, but it would be only the wavefront of the spectrally/temporally averaged beam. The CASSI system—and an advanced algorithm—allow to retrieve the spatio-spectral amplitude using the scrambled camera image. Since both the near-field and far-field are scrambled, phase-retrieval now allows one to find the spatio-spectral phase in addition to the amplitude. Finally, after combining with the spectral phase measurement of the FROG, the full spatio-temporal field is known.

**Fig. 1:**
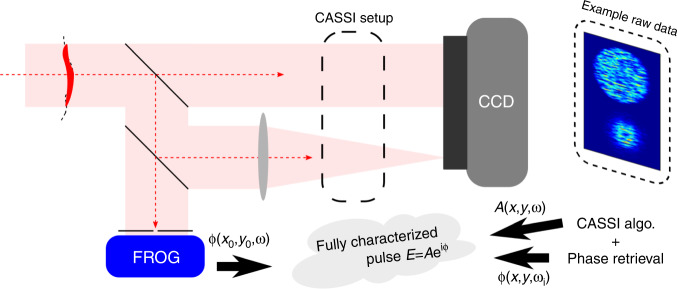
Basic layout of the COFT system and how it works. See the text for more details

Going beyond simply demonstrating COFT on a few ultrashort pulses, they also use it to diagnose a light-speed laser-matter interaction process. Focusing an ultrashort pump pulse in air produces an air-plasma that has structure traveling at the speed of light. A second ultrashort probe pulses propagates perpendicular to this interaction and experiences a spatio-temporal phase shift due to the air plasma. The researchers use COFT to measure the probe pulse and can resolve the light-speed refractive index structure in a single shot. Finally, in the last component of their work, the authors demonstrate an alternative way to implement COFT by using 3D spectral holography to calculate the spectrally-resolved wavefront rather than phase retrieval with a near-field/far-field set of images.

Despite being well-populated by successful techniques and even industrial products, the field of spatio-temporal characterization of ultrashort laser pulses experiences continued advances. The COFT technique presented in Tang, et al*.*^6^. represents a significant advance, which along with other recent work based on alternative inspirations from hyperspectral imaging^9^ demonstrates exciting future prospects. These advances in characterization will allow for continuing to improve the understanding of cutting-edge laser systems and exotic light-shaping techniques, and will be important for applications like secondary particle or X-ray generation from intense light-matter interaction, or processing of materials with ultrashort pulses.
